# Stability of vertical dimension following total arch intrusion

**DOI:** 10.1186/s12903-023-02842-1

**Published:** 2023-03-22

**Authors:** Dong-Ok Kang, Hyung-Seog Yu, Sung-Hwan Choi, Seong-Taek Kim, Hwi-Dong Jung, Kee-Joon Lee

**Affiliations:** Department of Orthodontics, Institute of Craniofacial Deformity, College of Dentistry, Yonsei University, 50‑1 Yonsei‑ro, Seodaemun‑gu, Seoul, 03722 Korea

**Keywords:** Total arch intrusion, Long face, Miniscrew implant, Stability, Relapse

## Abstract

**Background:**

The purpose of this study is to evaluate stability of vertical dimension following total arch intrusion using miniscrews by measuring the change during treatment and relapse amount after more than one year of retention.

**Methods:**

Thirty patients (6 men, 24 women) were included in this study. Lateral cephalographs were taken with conventional radiography at the start of treatment (T0), after treatment (T1), and at least one year after treatment (T2). The evaluation was performed by measuring changes of selected parameters during treatment and the extent of relapse after more than one year.

**Results:**

During total arch intrusion treatment (T1-T0), anterior and posterior teeth intruded significantly. The mean vertical distance between the maxillary posterior teeth and palatal plane was reduced by 2.30 mm (*P* < 0.001). The mean vertical distance between the maxillary anterior teeth and palatal plane was reduced by 2.04 mm (*P* < 0.001). The anterior facial height was also reduced by 2.70 mm (*P* < 0.001). During retention period (T2-T1), the vertical distance between the maxillary anterior teeth and the palatal plane significantly increased by 0.92 mm (*P* < 0.001). The anterior facial height increased by 0.81 mm (*P* < 0.01).

**Conclusions:**

Anterior facial height significantly decreases after treatment. During retention period, relapse of AFH and maxillary anterior teeth observed. There was no correlation between initial amount of AFH, mandibular plane angle, or SNPog and posttreatment AFH relapse. However, there was a significant correlation between the amount of intrusion of anterior and posterior teeth achieved by the treatment and the extent of relapse.

## Background

The correction of vertical skeletal discrepancy, such as open bite, gummy smile, lip incompetency and long face, has been a subject of orthodontic treatment. Orthognathic surgery has been recognized as a general method for vertical adjustment of long facial patterns, such as reducing anterior facial height and correcting a gummy smile. Rolf berg [1] analyzed 264 cases which completed the treatment, and reported that surgery is an indication for patients who have long face with mandibular clockwise rotation.

It has been reported about the stability of patients who had a long face and treated with orthognathic surgery. The long face can be improved by repositioning the maxilla upward through Le fort I surgery. Several studies have revealed that patients with a long face with a large mandibular plane angle showed more condylar resorption after surgery than that of patients with short face [2–4]. Mobarak et al. [5] reported the postoperative response of patients with skeletal class II malocclusion, and reported that the frequency and amount of relapse in the long face were greater and occurred in a long-lasting pattern. Conversely, there is also a study that the preoperative facial pattern is not related to condylar resorption and the condylar change is related to the amount of mandibular advancement [6].

Recently, tooth intrusion treatment has become possible with temporary anchorage devices (TADs) [7, 8]. The biomechanics and results of treatments using TADs are simpler than those of traditional treatment methods. As intrusion treatment became feasible with TADs, it was performed to intrude only one tooth [9]. Subsequently, segmental intrusion of the posterior teeth became clinically practicable, and non-surgical treatment of the open bite has been attempted [10]. Furthermore, anterior teeth intrusion or even total arch intrusion is required in some cases. In this case, both anterior and posterior teeth should be intruded unlike segmental intrusion or combined extrusion and intrusion. It became possible to intrude total arch, and orthodontic treatment of long face without surgery was perfomed [11].

The use of orthodontic miniscrews is more effective for anterior or posterior teeth intrusion than other orthodontic intrusion treatment methods [10, 12–14]. Biomechanical approach for intruding the entire arch accompanied by total arch distalization by using several miniscrews between teeth is being achieved. The two force vectors cause the direction of the force to pass through the center of resistance of the entire maxillary arch, causing intrusion and distalization of the entire dentition without changing the occlusal plane [15].

A study on posterior segmental intrusion was conducted to introduce treatment methods and results, and to show long-term stability [10]. However, the results, effectiveness, and long-term stability of total arch intrusion have not yet been reported.

The purpose of this study was to evaluate the stability of the vertical dimension following total arch intrusion using miniscrews by measuring the change during treatment and the amount of relapse after more than one year of retention.

## Methods

Total arch intrusion was performed using miniscrews in patients with malocclusion who visited the Department of Orthodontics, Yonsei University Dental Hospital, chiefly complaining of long face, gummy smile, or lip incompetency.

Patients who met the following criteria were selected from among those who had a maintenance period of one year or more: 1)high Frankfort-mandibular plane angle(FMA) (> 28.6˚) and 2)Skeletal Class I or Class II discrepancy (anteroposteriorly).

This study was conducted in full compliance with the Declaration of Helsinki. This study got exempt for ethical approval and review and for informed consent by the Institutional Review Board of Yonsei Dental Hospital (IRB No. 2–2022-0002).

**Fig. 1:**
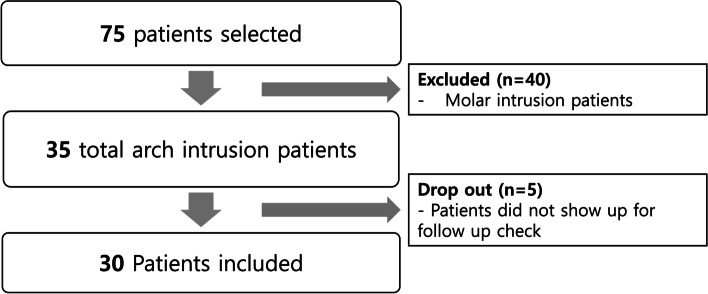
Study flow chart

Thirty patients (6 men, 24 women) met these criteria and were included in this study (Fig. 1). Eighteen of these patients had premolar extraction and twelve of these patients did not have.

The mean age at the start of treatment was 23.1 years (range, 14–44 years) and the mean treatment period was 34.6 $\pm$ 9.3 months (range, 10–51 months). The mean maintenance period was 3.9 $\pm$ 2.7 years (range, 1–11 years). All patients were given lingual fixed retainers; premolar extraction patients applied them between the premolars, and non-extraction patients applied them between the canines. In addition, they used usual circumferential retainers for maintenance; the retainers were applied on both the maxilla and mandible for the entire day during the first 6 months after treatment and only at night after that.

**Fig. 2:**
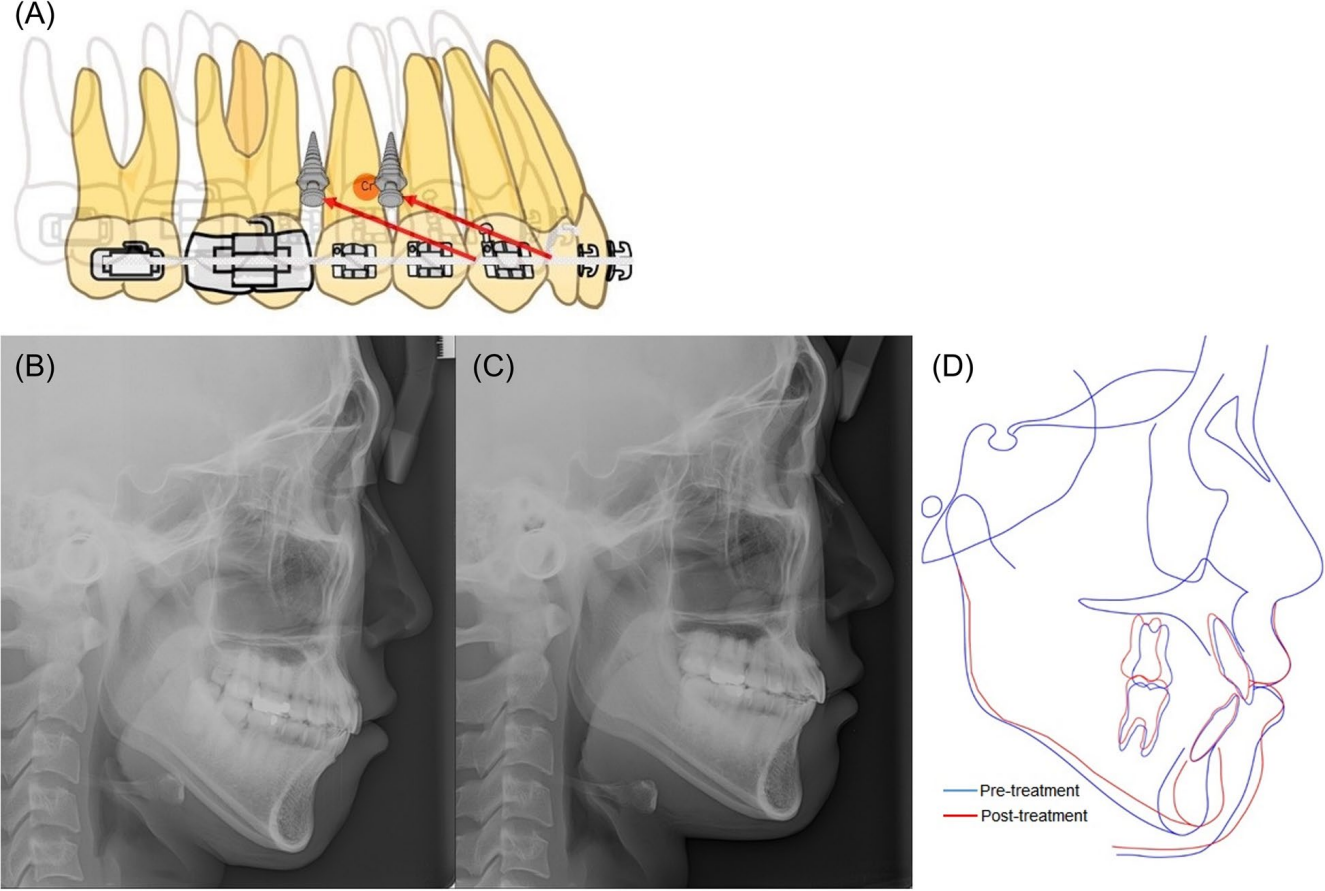
Total arch intrusion. **A** Biomechanics for total arch intrusion by using miniscrew implants, Lateral cephalograms of **B** pre-treatment, **C** post-treatment and **D** superimposition

**Fig. 3:**
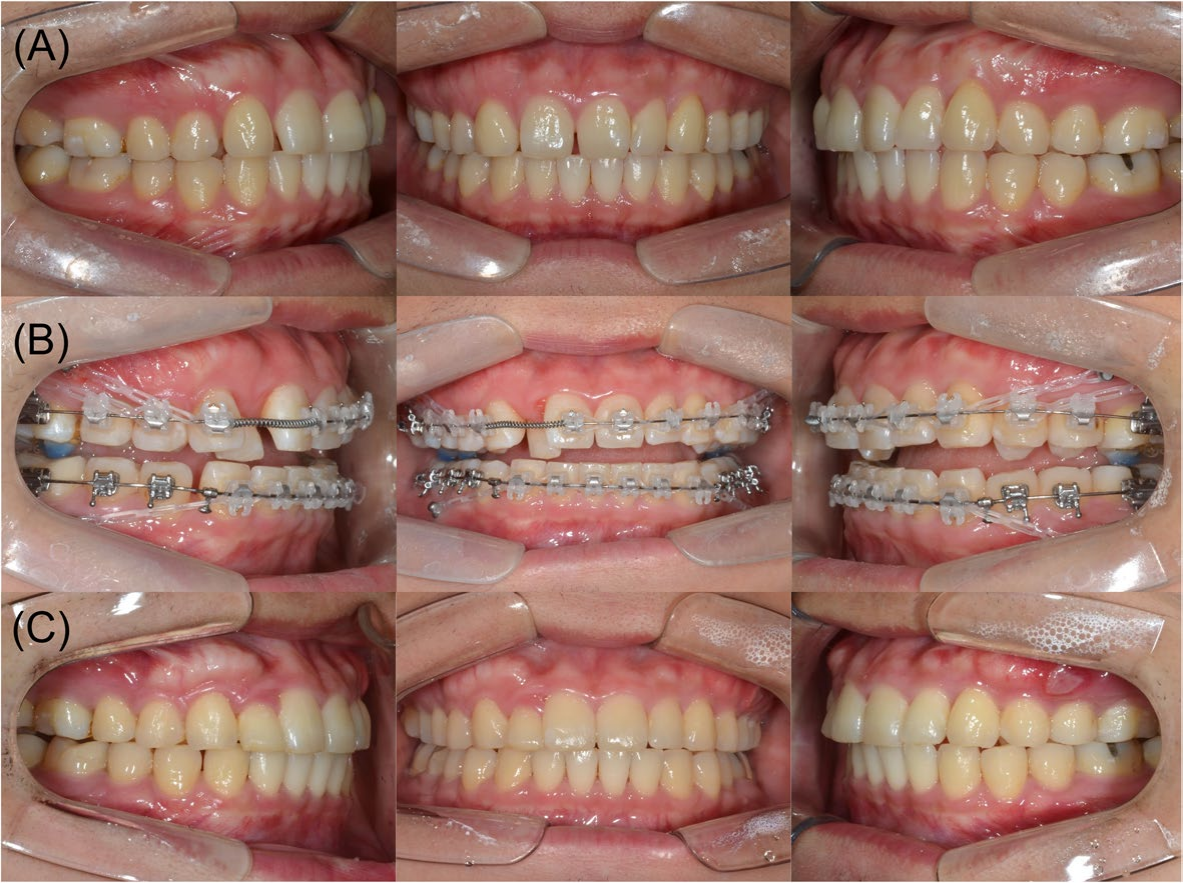
Intraoral photographs for case **A** pre-treatment, **B** mid-treatment, and **C** post-treatment

The following method was used for total arch intrusion. Miniscrews were placed on the buccal side between the roots of the maxillary first premolar and the second premolar, and between the roots of the maxillary second premolar and the first molar. After 3 to 4 weeks, an intrusive force was directly applied on the total arch using elastomeric chains (Figs. 2 and 3).

We use self-ligating ceramic brackets (Clippy C; Tomy) with an 0.018-in slot. Miniscrews with a diameter of 1.8 mm and length of 7.0 mm were used. Total arch intrusion force was 100–150 g for each miniscrew. Force was applied on a 0.016 × 0.022-in rectangular stainless steel archwire. The sample was treated under the supervision of a single practitioner (K.-J.L.).

In 20 of the 30 patients, mandibular miniscrews were additionaly inserted between the roots of the mandibular second premolar and first molar to prevent compensatory extrusion.

Lateral cephalographs were taken at the Department of Oral and Maxillofacial Radiology, Dental Hospital of Yonsei University College of Dentistry by using Cranex3 + (Soredex Orion corp, Helsinki, Finland), and saved in digital imaging and communications in medicine (DICOM) file format in the Picture Archiving Communication System (PACS; Infinitt Co, Ltd, Seoul, Korea). PACS digitizes and stores radiographic images from individual hospitals.

Some cephalometric radiographs taken before the introduction of PACS were scanned with a Diagnostic Pro Plus (Vidar Systems, Herndon, VA) scanner, and measurements were corrected and uploaded to PACS to be included in the study.

**Fig. 4:**
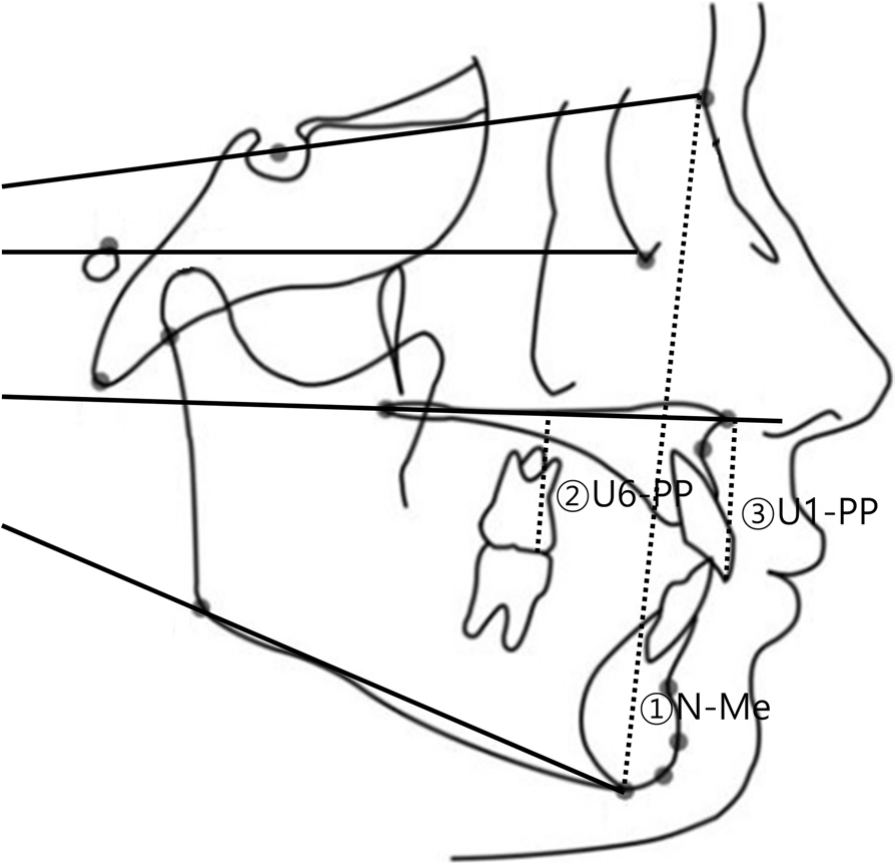
Cephalometric measurements: 1. N-Me: anterior facial height (AFH); 2. U6-PP: perpendicular distance from the mesiobuccal cusp of the maxillary first molar to the palatal plane; 3, U1-PP: perpendicular distance from the maxillary incisor edge to the palatal plane

Lateral cephalographs were taken with conventional radiography at the start of treatment (T0), after treatment (T1), and at least one year after treatment (T2). Lateral cephalographs were traced through the midpoint of the left and right structures. The measurements were corrected by considering a 110% magnification rate. Cephalometric landmarks, reference planes (Fig. 4), and cephalometric variables (Table 1) were used based on commonly used analyses.

**Table 1:** Cephalometric variables before treatment (T0), after treatment (T1), and at least one year after treatment (T2)

	*T0*		*T1*		*T2*	
*Variable*	*Mean*	*SD*	*Mean*	*SD*	*Mean*	*SD*
Skeletal						
ANB (°)	6.11	2.99	5.17	2.90	5.31	2.82
FMA (°)	35.35	5.08	33.65	5.65	34.34	5.64
SN-GoMe (°)	45.21	6.14	43.10	6.18	43.83	6.37
SNPog (°)	73.86	4.49	74.88	4.76	74.76	4.91
AFH (mm)	138.77	5.81	136.06	5.63	136.88	5.69
Dental						
IMPA (°)	96.00	5.72	89.83	7.14	92.23	6.72
U6-PP (mm)	27.39	2.07	25.09	2.30	25.42	2.09
U1-PP (mm)	35.16	2.22	33.12	2.23	33.94	2.06
L1-MP (mm)	48.60	3.23	46.28	3.05	46.70	3.11

The amount of vertical movement in the anterior teeth was measured by the amount of change in U1-PP, and the amount of vertical movement in the posterior teeth was measured by the amount of change in U6-PP.

### Statistical analysis

The lateral cephalographs stored in the PACS in DICOM file format were analyzed using V-ceph software (version 3.5, CyberMed, Seoul, Korea) to measure the landmarks and reference points.

For consistency, the measurement and analysis of all lateral cephalographs were performed by the same investigator (D.-O.K.). Once a week, to verify and reduce systematic errors, a number of reference points were randomly selected to remeasure and reanalyze the dimensions using paired *t* tests. No significant difference was noted between the measurements (*P* < 0.05).

The Shapiro-Wilk test was performed to verify normal distribution, the paired *t* test to compare variables between T0 and T1, and between T1 and T2, and the Pearson’s correlation analysis to examine their correlations.

## Results

The mean measurements for selected cephalometric variables from T0 to T2 are shown in Tables 1 and 2, and the correlation coefficients between the variables are presented in Tables 3, 4, and 5.

**Table 2:** Mean changes in the cephalometric variables at different time intervals

	*ΔT1-T0*^*a*^			*ΔT2-T1*^*b*^		
*Variable*	*Mean*	*SD*	*Sig*	*Mean*	*SD*	*Sig*
Skeletal						
ΔANB (°)	-0.95	0.97	***	0.14	0.52	NS
ΔFMA (°)	-1.70	1.82	***	0.70	1.64	*
ΔSN-GoMe (°)	-2.11	1.36	***	0.73	1.17	**
ΔSNPog (°)	1.03	1.04	***	-0.12	0.91	NS
ΔAFH (mm)	-2.70	1.37	***	0.81	1.37	**
Dental						
ΔIMPA (°)	-6.16	7.33	***	2.40	2.15	***
ΔU6-PP (mm)	-2.30	1.29	***	0.33	1.50	NS
ΔU1-PP (mm)	-2.04	1.66	***	0.83	0.92	***
ΔL1-MP (mm)	-2.33	1.83	***	0.42	0.89	*

Negative values represent decreases during treatment or retention; positive values represent increases during treatment or retention

*Sig* significance, *NS* Not significant

^*^P < 0.05; **P < 0.01; ***P < 0.001

^*a*^*ΔT1-T0,* change from initial evaluation to immediately after treatment

^*b*^*ΔT2-T1,* change during at least one year of retention;

**Table 3:** Correlation coefficients between cephalometric variables (T1-T0)

*Variable 1*	*Variable 2*	*R*	P	*Sig (2-tailed)*
U6-PP	FMA	0.085	0.657	NS
U6-PP	SNPog	-0.038	0.841	NS
U6-PP	SN-MP	0.077	0.685	NS
U6-PP	AFH	0.126	0.505	NS
U1-PP	FMA	0.240	0.201	NS
U1-PP	SNPog	-0.092	0.628	NS
U1-PP	SN-MP	0.137	0.469	NS
U1-PP	AFH	-0.103	0.589	NS
AFH	FMA	0.228	0.226	NS
AFH	SNPog	-0.464	0.010	*
AFH	SN-MP	0.691	< .001	***
FMA	SNPog	-0.267	0.153	NS
FMA	SN-MP	0.455	0.012	*
SNPog	SN-MP	-0.679	< .001	***

*T0* before treatment, *T1* after treatment, *R* Pearson correlation coefficient, *Sig* Significance, *NS* Not significant

^*^P < 0.05; **P < *0.01*; ***P < 0.001

**Table 4:** Correlation coefficients between pretreatment measurements (T0) and changes in anterior facial height (AFH: T2-T1)

*Variable 1* *(T0)*	*Variable 2* *(T2-T1)*	*R*	P	*Sig (2-tailed)*
AFH	AFH	-0.067	0.725	NS
SN-MP	AFH	0.120	0.528	NS
SNPog	AFH	-0.155	0.415	NS

*T0* before treatment, *T1* after treatment, *T2* at least one year after treatment, *R* Pearson correlation coefficient, *Sig* Significance, *NS* Not significant

**Table 5:** Correlation coefficients between T1-T0 variables and T2-T1 variables

*Variable 1* *(T1-T0)*	*Variable 2* *(T2-T1)*	*R*	P	*Sig.(2-tailed)*
U6-PP	U6-PP	-0.416	0.022	*
U1-PP	U1-PP	-0.575	< .001	***
AFH	AFH	-0.043	0.822	NS

*T0* before treatment, *T1* after treatment, *T2* at least one year after treatment, *R* Pearson correlation coefficient, *Sig* Significance, *NS* Not significant

^*^P < 0.05; ***P < 0.001

During total arch intrusion treatment, the anterior and posterior teeth intruded significantly. The mean vertical distance between the maxillary posterior teeth and the palatal plane was reduced by 2.30 mm (*P* < 0.001). The mean vertical distance between the maxillary anterior teeth and the palatal plane was reduced by 2.04 mm (*P* < 0.001). This decreased the ANB angle difference by 0. 95°(*P* < 0.001), the SN-GoMe angle by 2.11°(*P* < 0.001), and the anterior facial height by 2.70 mm (*P* < 0.001) (Tables 1 and 2).

During retention periods, the FMA angle increased by 0.70°(*P* < 0.05), the SN-GoMe angle by 0.73°(*P* < 0.01), and the anterior facial height by 0.81 mm (*P* < 0.01). Among the dental changes during this period, the vertical distance between the maxillary anterior teeth and the palatal plane significantly increased by 0.92 mm (*P* < 0.001) (Tables 1 and 2).

While analyzing the correlations between variables, we verified that AFH correction was induced by counterclockwise rotation of the mandible after the total arch intrusion treatment. The changes in treatment (T1-T0), of the SNPog (*P* < 0.05) and SN-MP (*P* < 0.001) were highly correlated with the change in AFH (Table 3).

By analyzing the correlations between the severity of the initial condition and the amount of AFH relapse, we found no significant correlation between the initial AFH, SN-MP, or SNPog and the amount of relapse (Table 4).

The correlation between the extent of the correction achieved by the treatment and the extent of relapse after at least one year of retention was also examined. The extent of intrusion performed on the maxillary anterior or posterior teeth was significantly correlated with the extent of relapse, whereas AFH reduction was not significantly correlated with relapse (Table 5).

The relapse rate was calculated. A total of 40.69% of the vertical distance between the maxillary incisors and palatal plane and 30.00% of the AFH relapsed after at least one year of retention. The vertical distance between the maxillary molars and the palatal plane did not significantly relapse (Table 6).

**Table 6:** Relapse rate of U1-PP and anterior facial height (AFH)

*Variable*	*Relapse rate (%)* *([T2-T1]/[T1-T0])*
U6-PP (mm)	NS
U1-PP (mm)	40.69
AFH (mm)	30.00

*U6PP* Vertical distance between maxillary molar mesial cusp tip and palatal plane, *U1PP* Vertical distance between maxillary incisor tip and palatal plane, *NS* Not significant

**Fig. 5:**
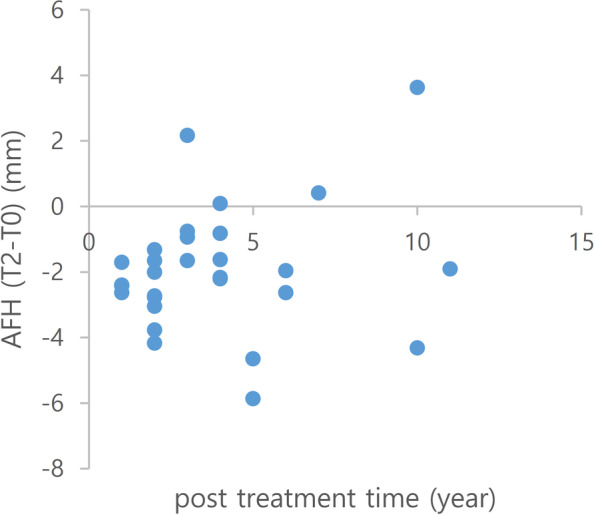
Changes in the anterior facial height(*AFH*) during the treatment and retention periods (T2-T0) according to post treatment time. *T0*, Before treatment; *T2*, at least 1 year after treatment

There was no significant relationship between the maintenance period after treatment and the relapse amount of anterior facial height (Fig. 5).

## Discussion

As a method for vertical correction of skeletal long face, orthognathic surgery has been recognized as a general method to control it. There are studies on the stability of long face patients treated with orthognathic surgery. Several studies have revealed that long-face patients with a large mandibular plane angle showed more condylar resorption after surgery than short-face patients [2–4]. Mobarak et al. reported the postoperative response of patients with skeletal class II malocclusion and reported that the frequency and amount of relapse in long-face patients were greater and occurred in a longer-lasting pattern [5].

The advent of the intraosseous anchor made posterior segmental intrusion possible. It made possible to treat an anterior open bite in adults without excessive extrusion of the anterior teeth, which improved the esthetics of the face. Sassouni [16] and Schudy [17] stated that posterior intrusion is the optimal treatment method as it can obtain stable treatment results and improve the long face with a skeletal anterior open bite.

Subsequently, it also became possible to intrude the total arch. Total arch intrusion can be chosen as a treatment option in hyperdivergent cases displaying severe lip protrusion and/or gummy smile [18]. Following clinical evidences support the idea that monocortical-type miniscrews can displace the whole dentition. Sugawara et al. [19, 20] introduced the use of miniplates for respective maxillary or mandibular molar without causing undesired movement of incisors. Related to this, simultaneous incisal and molar movement using interradicular miniscrews placed between the 2nd premolar and the 1st molar was proposed by others, eliminating the need for incisor retraction subsequent to the molar distalization [21, 22].

Upadhay et al. [23, 24] studied the clinical outcome of en masse retraction of the anterior teeth following premolar extraction using bimaxillary interradicular miniscrews in randomized controlled clinical trial. Interesting finding was the reduction of the mandibular plane angle in the mini-implant group, which was not evident in the conventional retraction group. It was suggesting an intrusive effect.

Furthermore, Bechtold et al. [15] found intrusion of the entire dental arch during distalization of the dental arch using dual interradicular miniscrews in the maxilla. An additional force vector created by interradicular miniscrews positioned between the 1st and the 2nd premolars changed the relationship between the imaginary center of resistance of the entire arch and the lines of force. It caused simultaneous upward and backward displacement of the entire dental arch.

In total arch intrusion, the pattern of displacement is affected by the location of force vector because the distance between the center of resistance and the line of force changes greatly. Even if the center of resistance of the entire arch is localized by experimental methods, it is still difficult to pinpoint the center of resistance in various case. Therefore, it is recommended to use dual miniscrews for predictable total arch intrusion, (Fig. 2) to stabilize the dental arch during intrusion without tilting, and also to have the intrusion force efficiently distributed throughout the dental arch [18].

By using dual miniscrews between the teeth, the two force vectors cause the direction of force to pass through the center of resistance of the entire maxillary arch, causing intrusion and distalization of the entire dentition without changing the occlusal plane [15]. Therefore, clinicians can control the ratio of intrusion and distalization of the entire dentition by adjusting the force vector direction. When total arch distalization occurs simultaneously with total arch intrusion, it is effective in correcting a protruded lip, gummy smile, and lip incompetency, improving smile esthetics [11].

Skeletal changes during total arch intrusion include mandibular counterclockwise rotation, which results in a decrease in the mandibular plane angle and anterior facial height (Table 2). Kuroda et al. [14, 25] and Sugawara et al. [26] also reported that posterior intrusion using skeletal anchorage caused counterclockwise rotation of the mandible, reducing the lower facial height and improving the anteroposterior jaw relationship. In our study, Skeletal changes that occurred during the maintenance period of at least one year after the end of treatment showed a statistically significant relapse (Table 2). Intruded maxillary arch was extruded during the retention period, resulting in an increase in the mandibular plane angle and anterior facial height (Table 2).

After treatment, the maxillary incisors and molars were intruded, and the anterior facial height decreased significantly (Table 2). The anterior facial height was reduced by 2.70 mm (*P* < 0.001). This had an effect on the improvement of the facial length. It was found that counterclockwise rotation of the mandible caused a reduction in the mandibular plane angle, which improved facial length. This can also be seen from the fact that there was no correlation between the amount of anterior and posterior intrusion and the reduction in anterior facial length (Table 3). In addition, there was no significant difference in the amounts of intrusion of the maxillary anterior and posterior teeth and in the reduction of AFH during treatment between the groups treated with and without premolar extraction.

**Fig. 6:**
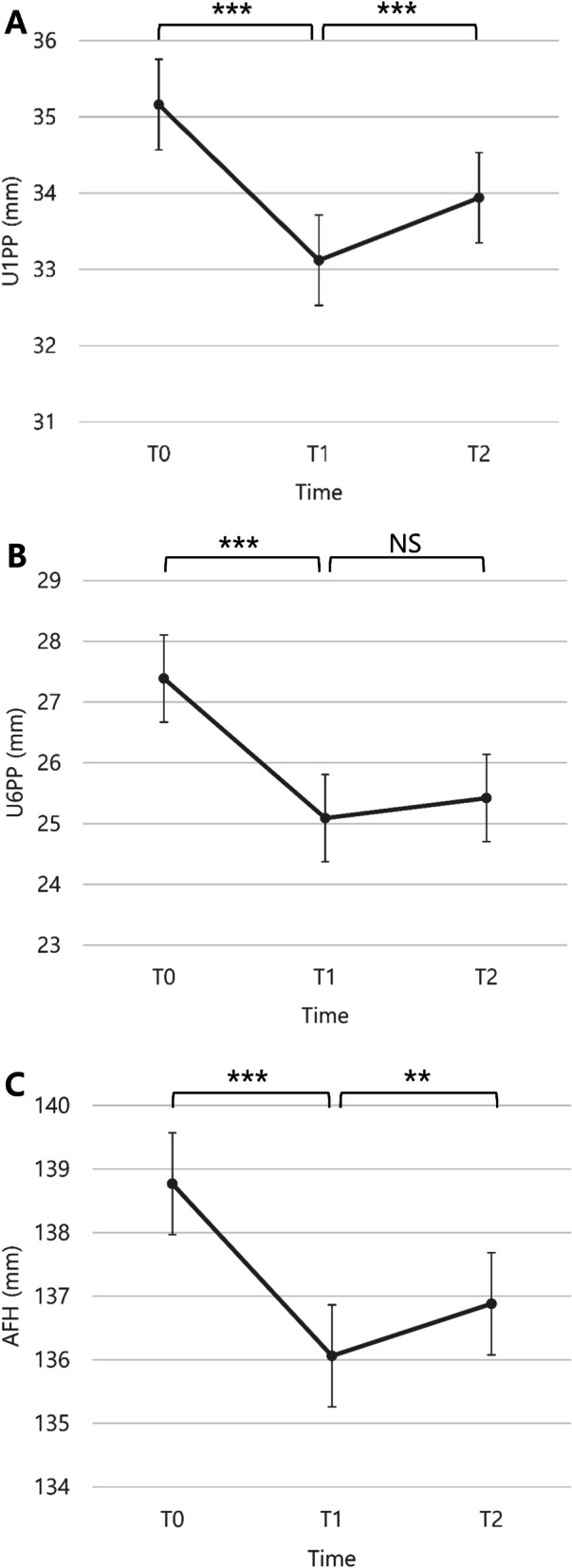
**A** Changes in the vertical distance between the maxillary incisor tip and the palatal plane(*U1PP*). **B** Changes in the vertical distance between the maxillary first molar tip and the palatal plane(*U6PP*). **C** Changes in the anterior facial height(*AFH*) during the treatment and retention periods. *T0*, Before treatment; *T1*, after treatment; *T2*, at least one year after treatment; *NS*, not significant. **P < 0.01; ***P < 0.001

Statistically significant relapse of facial length and maxillary anterior intrusion occurred during the maintenance phase of at least one year after the end of treatment (Table 2 and Fig. 6).

In this study, miniscrews were placed on the buccal side between the roots of the maxillary first premolar and the second premolar, and between the roots of the second premolar and first molar. After 3–4 weeks, an intrusive force was directly applied on the total arch using elastomeric chains. When a continuous arch wire is used, the target segment is the whole arch, and the center of resistance can be localized around the premolar (Fig. 2).

Reitan [27] reported that intruded teeth are more stable than extruded teeth because occlusal force is applied continuously. In our study, the stability of the total arch intrusion was evaluated, and the relapse of the posterior intrusion was significantly smaller than that of the anterior region (Table 3). It can be inferred that the posterior region is more stable because it continuously receives greater occlusal force. The stability of the anterior facial height (AFH) was evaluated in our study to determine the stability of non-surgical long-face treatment. The AFH relapse rate was 30.00%. More than half of the reduction amount of AFH during treatment in our study was maintained, so total arch intrusion is effective as a non-surgical treatment method for skeletal long face. It would be more desirable to overcorrect in consideration of the relapse.

An effective method of retention after intrusion treatment has not been introduced yet. Therefore, this study used the usual maintenance method, and the extent of relapse was assessed when maintained by the usual method. Considering the soft tissue recovery time for orthodontic treatment, retentions are needed all day long immediately after the removal of fixed orthodontic appliances for 3–4 months, after which they can be used partially during the day [28].

Unlike the previous notion [10], no significant relationship was observed between the maintenance period after treatment and the extent of relapse of the anterior facial height. Alternatively, the extent of relapse could be related to individual’s characteristics. Therefore, conducting further studies with a large number of patients and longer retention periods is important.

This study included patients aged 14 years or older. On average, after the age of 14, there is no dramatic skeletal growth beyond the maximum growth period [29, 30].

In this study, there was no correlation between the mandibular plane angle and SNPog angle before treatment and the amount of relapse of the anterior facial height after treatment (Table 4).

Regarding the correlation between the amount of change by treatment and the amount of relapse, the change of maxillary incisors and molars during treatment showed a statistically significant correlation with the amount of their relapse (Table 5). The amount of their relapse was proportional to the maxillary anterior and posterior intrusion achieved during treatment.

Lee et al. [31] reported that the relapse rate of the maxillary posterior intrusion in the maintenance period of one year after the end of treatment was 10.36%. This result showed superior stability compared to the study by Sugawara et al. [26] that reported a 30% relapse rate of mandibular posterior intrusion in the maintenance phase of one year after the end of treatment. In our study, in the maintenance phase of at least one year after the end of treatment, 40.69% of maxillary anterior intrusion and 30.00% of anterior facial height changes relapsed (Table 6). The vertical distance between the maxillary molars and the palatal plane did not significantly relapse. The relapse shown in this study can reduce the need for surgery; therefore, it is considered a good treatment option for patients with mild or moderate skeletal long face. Many literatures about the stability of long face treatment mention the small size of the study subject, short maintenance period, and lack of a control group as limitations of the study. These limitations also apply to this study and it is considered that a larger number of patients will be added in a prospective study with a quantified treatment method in the future. Furthermore, it is important that follow-up studies with longer retention periods should be continued.

## Conclusion

This study aimed to evaluate the stability of the vertical dimension following total arch intrusion treatment. This evaluation was performed by measuring changes in selected parameters during treatment and the extent of relapse after more than one year. The following conclusions were drawn:

1. Skeletally, counterclockwise rotation of the mandible occurs after treatment, which in turn improves skeletal long face. The anterior and posterior teeth intruded significantly during treatment.
2. Anterior facial height significantly decreases after treatment.
3. During the retention period, relapse of AFH and maxillary anterior teeth were observed.
4. There was no correlation between the initial amount of AFH, mandibular plane angle, or SNPog, and post-treatment AFH relapse. However, there was a significant correlation between the amount of intrusion of the anterior and posterior teeth achieved by treatment and the extent of relapse.

Total arch intrusion can be chosen as a treatment option in hyperdivergent cases displaying severe lip protrusion and/or gummy smile. If an appropriate retention method is applied during the maintenance phase, considering the initial skeletal configuration, muscle force, and influence of the tongue and soft tissues, it would effectively enhance the long-term stability of total arch intrusion treatment.

## Data Availability

All data generated or analyzed during this study are included in this published article.

## Abbreviations

AFH: Anterior facial height
SNPog: Tha angle between Sella, Nasion and Pogonion
SNMP: Sella-Nasion-Mandibular plane angle
IMPA: The angle between lower incisor axis and mandibular plane
U6-PP: Perpendicular distance from the mesiobuccal cusp of the maxillary first molar to the palatal plane
U1-PP: Perpendicular distance from the maxillary incisor edge to the palatal plane
L1-MP: Perpendicular distance from the mandibular incisor edge to the mandibular plane
FMA: The Frankfort-mandibular plane angle
PACS: Picture archiving communication system
DICOM: Digital imaging and communications in medicine
TAD: Temporary anchorage devices
